# Quantification of Myocardial Blood Flow by Machine Learning Analysis of Modified Dual Bolus MRI Examination

**DOI:** 10.1007/s10439-020-02591-0

**Published:** 2020-08-20

**Authors:** Minna Husso, Isaac O. Afara, Mikko J. Nissi, Antti Kuivanen, Paavo Halonen, Miikka Tarkia, Jarmo Teuho, Virva Saunavaara, Pauli Vainio, Petri Sipola, Hannu Manninen, Seppo Ylä-Herttuala, Juhani Knuuti, Juha Töyräs

**Affiliations:** 1 Diagnostic Imaging Center, Kuopio University Hospital, PO Box 100, 70029 KYS Kuopio, Finland; 2 Department of Applied Physics, University of Eastern Finland, Kuopio, Finland; 3 School of Information Technology and Electrical Engineering, The University of Queensland, Brisbane, Australia; 4 A.I. Virtanen Institute for Molecule Sciences, University of Eastern Finland, Kuopio, Finland; 5 Turku PET Centre, University Hospital and University of Turku, Turku, Finland; 6 Department of Medical Physics, Turku University Hospital, Turku, Finland; 7 Heart Center and Gene Therapy Unit, Kuopio University Hospital, Kuopio, Finland

**Keywords:** Magnetic resonance imaging, Myocardial perfusion imaging, Modified dual bolus method, Machine learning, Random forest, Support vector machine

## Abstract

Contrast-enhanced magnetic resonance imaging (MRI) is a promising method for estimating myocardial blood flow (MBF). However, it is often affected by noise from imaging artefacts, such as dark rim artefact obscuring relevant features. Machine learning enables extracting important features from such noisy data and is increasingly applied in areas where traditional approaches are limited. In this study, we investigate the capacity of machine learning, particularly support vector machines (SVM) and random forests (RF), for estimating MBF from tissue impulse response signal in an animal model. Domestic pigs (*n *= 5) were subjected to contrast enhanced first pass MRI (MRI-FP) and the impulse response at different regions of the myocardium (*n *= 24/pig) were evaluated at rest (*n *= 120) and stress (*n *= 96). Reference MBF was then measured using positron emission tomography (PET). Since the impulse response may include artefacts, classification models based on SVM and RF were developed to discriminate noisy signal. In addition, regression models based on SVM, RF and linear regression (for comparison) were developed for estimating MBF from the impulse response at rest and stress. The classification and regression models were trained on data from 4 pigs (*n *= 168) and tested on 1 pig (*n *= 48). Models based on SVM and RF outperformed linear regression, with higher correlation (*R*_SVM_^2^ = 0.81, *R*_RF_^2^ = 0.74, *R*_linear_regression_^2^ = 0.60; *ρ*_SVM_ = 0.76, *ρ*_RF_ = 0.76, *ρ*_linear_regression_ = 0.71) and lower error (RMSE_SVM_ = 0.67 mL/g/min, RMSE_RF_ = 0.77 mL/g/min, RMSE_linear_regression_ = 0.96 mL/g/min) for predicting MBF from MRI impulse response signal. Classifier based on SVM was optimal for detecting impulse response signals with artefacts (accuracy = 92%). Modified dual bolus MRI signal, combined with machine learning, has potential for accurately estimating MBF at rest and stress states, even from signals with dark rim artefacts. This could provide a protocol for reliable and easy estimation of MBF, although further research is needed to clinically validate the approach.

## Introduction

Myocardial blood flow (MBF) is an important parameter for diagnosing heart diseases or examining the state of the myocardium. Positron emission tomography (PET), the gold standard in the diagnostics of the myocardial perfusion, uses radioactive water (i.e., water including ^15^O) as a tracer for quantification of absolute MBF.^7^ However, the disadvantages of PET include radiation exposure and limited availability of radioactive water. Although the risk posed by radiation in PET is very often justified, minimizing the radiation dose is always beneficial. The main motivation behind the present study is the limited accessibility of PET. It is not accessible for all patients suffering from myocardial infarction, whereas MRI scanners are much better available. In addition, compared with PET, MRI provides more information during the same imaging session. MRI offers an opportunity to investigate the anatomy and the pump function of the heart. Certain MR imaging sequences yield parametric maps (for example, T_1_- or T_2_-map), which enable tissue characterization. MRI also offers the possibility to follow-up of response to therapy or progresison of disease. For example, change of lifestyle, medication and/or interventions may influence LV characteristics such as mass, volume, function and perfusion which all can be monitored with MRI.^19^ Monitoring of human patients is challenging with method that uses ionizing radiation. Furthermore, AI provides a powerful and fast method for analysis of MR images.^9^,^15^ The use of AI in biomedical applications is under very active research and it is becoming the standard in many (if not all) medical image processing tasks.^5^ Therefore, investigating the potential of AI for the present purposes is well justified and timely.

One MRI-based method for assessing myocardial perfusion is contrast agent-enhanced magnetic resonance imaging (MRI), which allows determination of parameters describing perfusion.^14^ While this method is promising for determination of MBF, it suffers from noise originating from imaging artefacts, potentially impeding accurate estimation of MBF from the MRI impulse response signal.^4^

To address this shortcoming, we employ machine learning methods, for the first time, for estimating MBF from MRI impulse response signal. Machine learning is an application of artificial intelligence (AI) where the aim is to enable a machine learning models to learn automatically from data without human intervention. The learning process begins with training the machine learning models using certain learning algorithms and data with known input and output values. The learning algorithm then generates a set of rules, based on inferences from the data. The result is a model, which defines the relationship between input and output. The model can then be used to predict outputs from new inputs. In general, machine learning represents a class of analytical and statistical methods capable of extracting important features from multivariate input data, including artefact-induced noisy data. In biomedicine, machine learning techniques may provide solutions to complex problems, allowing machine learning models to make predictions from large amounts of patient data. Clinical decision support systems (DSS) are in widespread use in medicine.^3^ In the field of medical imaging, machine learning has so far been used mainly for automated segmentation of images.^15^ More so, they are seeing increasing biomedical applications in areas where traditional approaches are limited, such as oncology,^12^ musculoskeletal^1^ and neuroscience research.^11^ Methods such as support vector machines (SVM) and random forest (RF) are among the most popular machine learning algorithms that have demonstrated potential for clinical applications, such as disease screening and diagnosis.^3^,^11^ Machine learning enables fast analysis of massive amount of data.

Support vector machine is a non-probabilistic machine learning method, which in contrast with probabilistic techniques, such as the Naïve Bayes, separates data across a decision boundary (plane) determined only by a small subset of the input data (predictors). The subset of input data that supports the decision boundary are the support vectors, and the remaining input data do not have any influence in determining the position of the decision boundary. Random forest is a machine learning algorithm that consists of a large number of individual decision trees operating as an ensemble. As the basic construct of RF, a decision tree allows observations to be split in a way that the resulting groups are as different from each other as possible, while the members of each subgroup are as similar to each other as possible. Each individual tree in the random forest outputs a prediction, e.g., class, and the class with the most votes becomes the model’s prediction.

In this study, we investigate and compare the capacity of SVM and RF for estimating MBF from tissue impulse response signal. For comparison, MBF was also determined using traditional approach based on linear regression. This study is based on the hypothesis that machine learning methods are capable of accurately estimating MBF from tissue impulse response signals, even from signal with artefact-induced noise, due to their capacity to extract relevant features from predictors during analysis. To test this hypothesis, we develop machine learning regression and classification models for predicting MBF from tissue impulse response signal and detecting samples with artefacts from their impulse response signals, respectively. In addition, we assess the relative effect of artefact-induced noise on model performance for predicting MBF from the tissue impulse response. Specifically, we applied these methods in a porcine model of myocardial ischemia with modified dual-bolus contrast agent-enhanced MRI examination.

## Materials and Methods

### Animal Model

All animal experiments were approved by the National Animal Experiment Board (license n:o ESAVI-2012-001932) and conform to the Directive 2010/63/EU of the European Parliament. Five female domestic pigs (age (mean ± SD) (3.5 ± 0.5) months and weight (35.6 ± 7.4) kg), were examined with MRI and PET. Local myocardial ischemia was induced using a constricted bare metal stent.^16^ Pigs were imaged within 12–24 days after the operation.

### PET Imaging Protocol

PET and MRI imaging were performed during the same session keeping the positioning unchanged between the modalities. Philips Ingenuity TF PET/MR (Philips, Amsterdam, the Netherlands) scanner was used for both PET and MRI imaging. Animals were anaesthetized, connected to a respirator and ventilated mechanically. Anesthesia was maintained with an intravenous infusion of Propofol (10 − 50 mg/kg/h, Propofol Lipuro, B. Braun Melsungen AG, Melsungen, Germany) combined with fentanyl 4 − 8 µg/kg/h (Fentanyl-Hameln, Hameln Pharmaceuticals GmbH, Hameln, Germany). Diastolic, systolic and mean arterial pressure and heart rate (HR) were recorded using a pressure transducer (TruWave, Edwards Lifesciences Corp., Irvine, CA, USA) connected to an anesthesia monitor (Datex Ohmeda S5, GE Healthcare Finland Oy, Helsinki, Finland).

First, a myocardial perfusion PET study with [^15^O]-water under pharmacologic stress was performed. Pharmacologic stress was induced with intravenous infusion of adenosine at a rate of 500 μg/kg/min, (Adenosin Life Medical, Life Medical Sweden AB, Stocksund, Sweden) combined with phenylephrine 5 μg/kg/min (Fenylefrin Abcur, Abcur AB, Helsingborg, Sweden) administered intravenously starting 2 min prior to PET imaging and continuing throughout the stress study to induce myocardial hyperemia. The [^15^O]-water (790 ± 74 MBq, range 628–879 MBq) (Radiowater Generator, Hidex Oy, Turku, Finland) was injected intravenously via the ear vein as a 15 s bolus. The dynamic scanning started at the same time as the injection. After that, the PET imaging was repeated at rest after the hemodynamics had returned to the base level.

### MR Imaging Protocol

For MRI, the pig was moved out from the PET gantry, and four-lead electrocardiogram electrodes for cardiac gating were attached. Subsequently, a cardiac array surface coil was positioned over the chest, and the pig was moved into the MRI scanner on the same table. The pigs were ventilated normally during the MR perfusion imaging. After the scout images were acquired, four-chamber and two-chamber cinematic series, perpendicular to each other, were acquired. The cinematic series were used to prescribe the imaging slices for first-pass contrast-enhanced myocardial perfusion imaging. Two parallel 8 mm slices with 16 mm gap were placed on the short axis of the left ventricle (LV), starting 16 mm from apex and continuing to mid-ventricular level. 2D saturation recovery segmented gradient recalled echo (T1-TFE) sequence was used for perfusion imaging. Imaging parameters were: flip angle = 20°, acquisition matrix = 92 × 128 and field of view = 350 × 350 mm; the TR and TE of the acquisition train were set to the shortest possible (1.6/3.3 ms), and the scan repetition time (heart rate dependent TR) was between 580 ms and 1380 ms, depending on the heart rate. Saturation recovery time was set to 150 ms. To correct the nonlinear relationship between the contrast agent concentration in blood and the MR signal intensity during the first-pass, a 0.05 mmol/kg tracer bolus of diluted contrast agent (gadoteric acid, Dotarem, 0.5 mmol/mL Guerbet LLC, Bloomington, IN, USA, dilution: 5 mL Dotarem + 100 mL 0.9% saline, concentration ratio: 5 mL/105 mL = 1/21) was manually injected into the ear vein as fast as possible, and thereafter 15 mL of saline was injected manually for flushing. Dynamic MR imaging was repeated continuously for every cardiac cycle during 60 consecutive heartbeats. Thereafter, the actual perfusion series was acquired in a similar fashion except that the contrast agent was administered without dilution. Dynamic imaging procedure, including calibration and actual perfusion series, was carried out for both stress and rest conditions similarly to PET perfusion imaging procedure.

### Image Analysis

**Figure 1:**
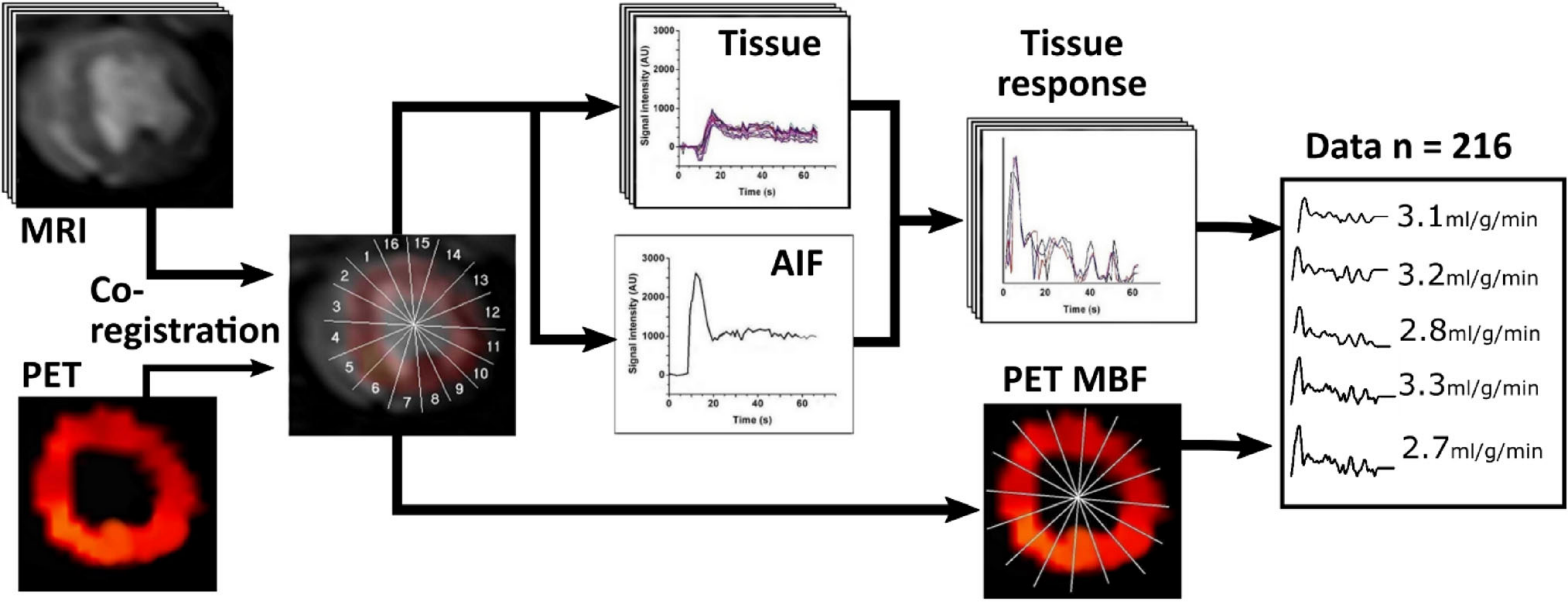
Procedure of the image analysis and data processing. First, PET and MR images were co-registered into same orientation. Then the myocardium was divided into ROIs. MBF values of ROIs were obtained from the PET image. Same ROIs were used to extract the SI–time curves of myocardium from MR images. The arterial input function (AIF) SI-time curve was acquired from the left ventricle blood pool. Next, AIF and MRI myocardium data were used to calculate the tissue impulse responses for each ROI. Data consists of all the tissue impulse responses and corresponding values of MBF from PET.

Some of the perfusion data investigated in this study has been previously analyzed using a model independent deconvolution method and reported.^6^ In that previous analysis, the signals containing dark rim induced noise had to be excluded. To further enhance the accuracy of estimation of MBF, the whole data including also signals containing artefacts is re-analyzed in the present study using linear regression, RF and SVM techniques. The procedure of the image analysis and data processing is schematically presented in Fig. 1.

**Figure 2:**
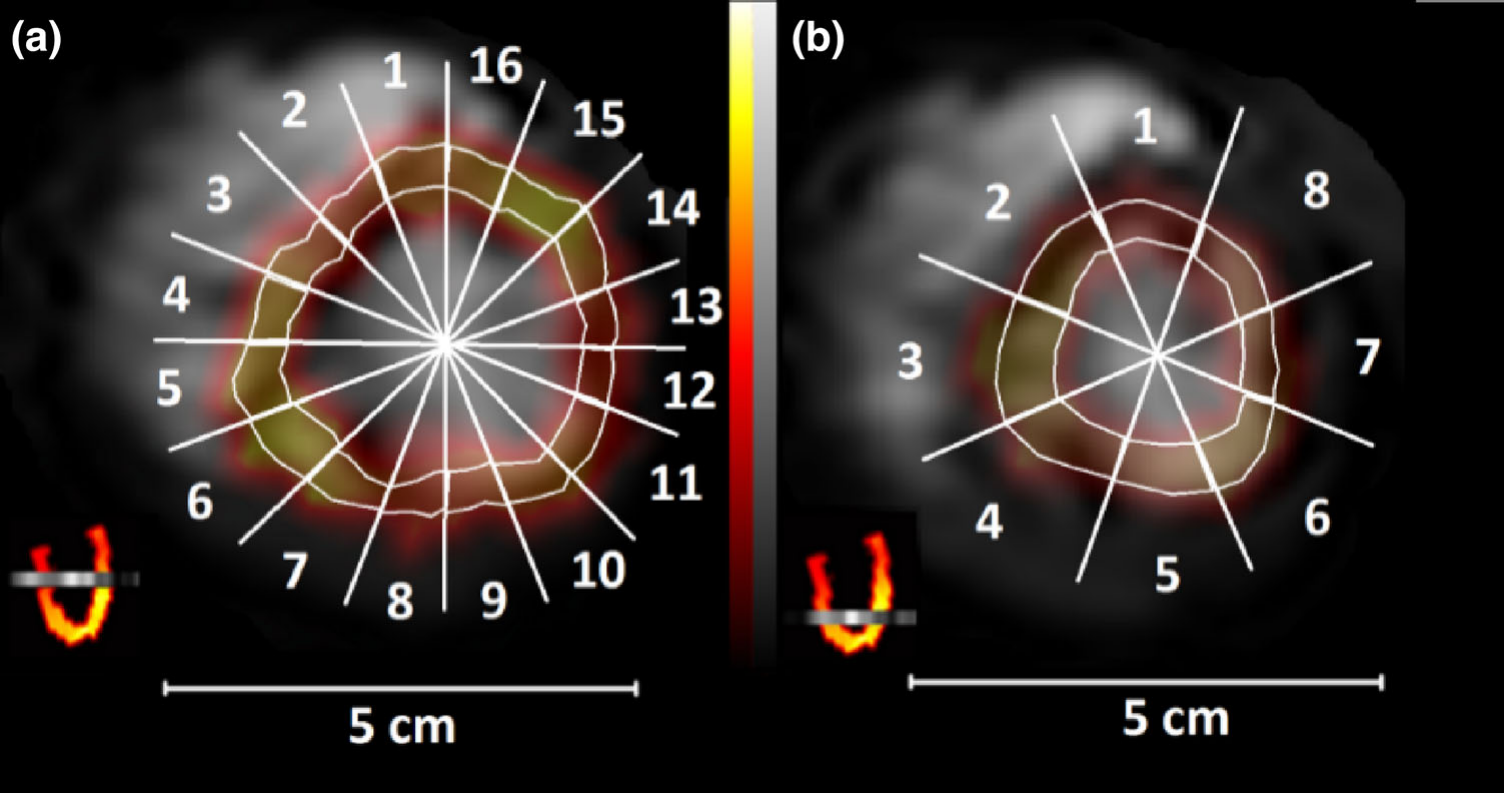
Co-registered short axis MRI- and PET-images with ROIs (delineated with grey lines). (a) A mid-ventricular slice and (b) an apical slice. Orientation of the MRI slices is presented in long axis images (inset in lower left corner). MRI images were acquired using a T1-TFE sequence. Contrast agent can be seen in the right and left ventricles.

Images were analyzed with the Carimas software (Version 2.9, Turku, PET centre, Finland 2014).^13^ At first, PET and MR images were co-registered, and regions of interest (ROIs) in the left ventricle and myocardium were delineated in MR images. The apical slice was divided into eight, and the mid-ventricular slice into sixteen similarly sized regions (Fig. 2).

2D ROIs were converted into 3D volumes of interest (VOI), and signal intensity (SI)–time curves for each VOI were obtained. SI–time curves of MR images were processed in Matlab (v. 2014b, The MathWorks, Natick, MA, USA). Next, the VOIs in MR images were copied into co-registered PET images, and values of MBF for each VOI were determined from the PET data.

PET data was analyzed using the Carimas software. A ROI covering the whole LV was applied to the dynamic imaging series in order to obtain myocardial time–activity curves (TAC). A cylindrical volume of interest located in the basal portion of the LV was applied for obtaining arterial input function. The segmental average LV MBF was determined based on [^15^O]-water images using the conventional single-compartment model.^7^

Some tissue enhancement curves of MR images had to be excluded from further analyses: the stress study of one pig (#3) failed technically and had to be rejected. Therefore, altogether 216 MR signals were included in the study for further analysis. A total of 103 tissue enhancement curves (48%) were found to be affected by dark rim artefact^4^ appearing usually in the septum area. If the SI of tissue enhancement curve dropped below the baseline at the arrival of the contrast agent (and subsequent increase of SI), the curve was labeled as being artefacted.

### Tissue Impulse Response

The tissue impulse response describes the ability of the system to transfer the contrast agent from capillary blood into the myocardium. The relationship between contrast agent concentration in blood and myocardium can be defined by convolution

1 $$C_{\text{t}} (t) = C_{\text{b}} (t) \otimes h(t),$$

where *C*_t_(*t*) is the MR contrast agent concentration in tissue, *C*_b_(*t*) is the contrast agent concentration in blood (i.e., the AIF) and *h*(*t*) is the tissue impulse response.^2^ Therefore, *h*(*t*) can be determined using deconvolution

2 $$h(t) = \frac{{C_{\text{t}} (t)}}{{C_{\text{b}} (t)}}$$

The tissue impulse response for each tissue curve was calculated using regularized model independent deconvolution.^8^ T_1_- and T_2_*-effects cause distortion to the arterial input function^17^ because of the nonlinear relationship between signal intensity and high contrast agent concentrations. This phenomenon occurs in AIF during the first pass, causing a blunted peak. To avoid errors in calculation of the perfusion parameters, correction procedure for the high concentration AIF was performed. The modified dual bolus correction procedure is fully described in our earlier paper.^6^

### Classification and Regression Models and Hyperparameter Tuning

**Figure 3:**
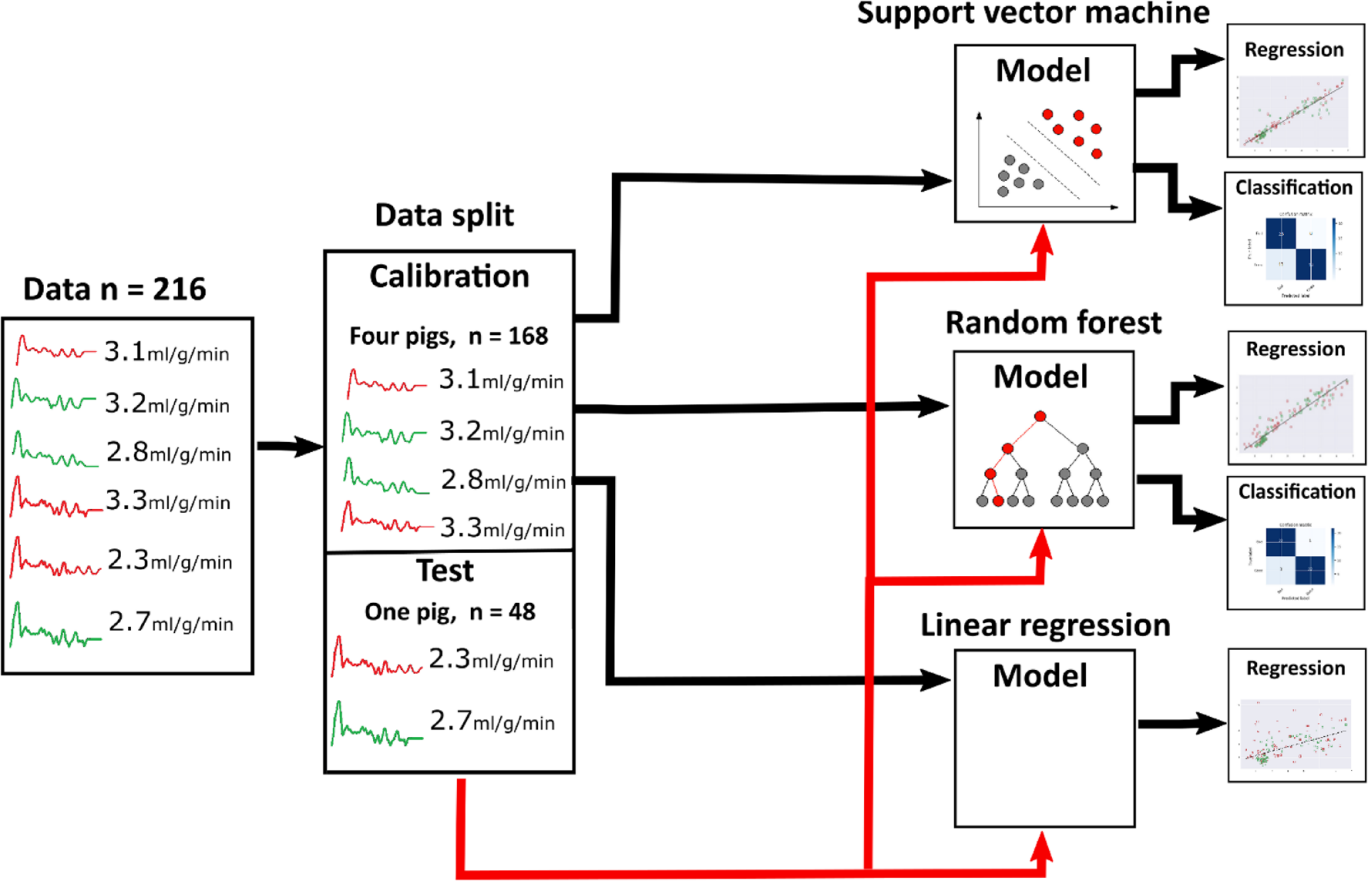
Analysis of the data. First, data is split to calibration and training sets. Calibration set is used to train the model (SVM, RF or linear regression). Test set is then used as a new input for testing the created model. Red data curves indicate the impulse responses with dark rim artefact-induced noise, and green ones without this noise.

Prior to analysis, the full dataset at rest and stress states, consisting of the tissue impulse response (predictors/independent variable), data integrity label (class) and reference MBF (dependent variable) values, was split into calibration and test sets, where the calibration set consisted of data from 4 pigs (*n*_total_= 168, *n*_rest_= 96, *n*_stress_= 72), and data from the remaining pig was used as an independent test set (*n*_total_= 48, *n*_rest_= 24, *n*_stress_= 24, ≈ 22%) for evaluating model performance (Fig. 3).

It is worth noting that data from the test set was not involved in model training or hyperparameter tuning, but only used for estimating model performance. The criteria for selection of the test set was that the test set variance does not exceed the variance of the calibration set. Since tissue impulse response signal may include artefact-induced noise, classification models based on SVM and RF were developed to discriminate between intact and artefact-induced noisy signal, that is, classifying the “*good*” (intact) or “*bad*” (artefacted). In addition, regression models for estimating MBF from tissue impulse response signals were developed using SVM, RF and traditional approach based on linear regression.

Since SVM and RF consist of multiple hyperparameters that define a model and affect its performance on an independent data, e.g., affecting the decision boundary of SVM, it is essential to find the best combination of hyperparameters that maximizes the relationship between the tissue impulse response and label (for classification) or MBF (for regression), thus optimizing model performance and generalization.^10^ To determine the optimal hyperparameter combination, a ‘fit’ and ‘score’ method was employed using the grid search algorithm with 5-fold internal cross-validation. In this method, a parameter grid for each estimator (SVM and RF) is first defined, a model is fitted on a part of the calibration dataset using a specific hyperparameter combinations, and then scored on the remaining part of the dataset. This is repeated for all possible combinations of the pre-defined parameters. Thus, the algorithm performs an exhaustive cross-validated search over the grid of specified parameter values for each estimator, and a model is fitted and scored for each specified parameter value. For SVM, the relevant hyperparameters tuned include: kernel, regularization constant (*C*), gamma (parameter of a Gaussian Kernel) and degree, while for RF, the hyperparameters include: number of estimators, criterion, max depth (the depth of each tree in the forest), and max features (the number of features to consider when looking for the best split).

Based on the number of pre-defined hyperparameters, a total of 2500 and 10800 models were fitted for SVM and RF, respectively. This approach was adopted for hyperparameter tuning in classification and regression, and the best model was selected based on the cross-validated score. After hyperparameter tuning and model selection, model performance was evaluated using the independent test dataset. For regression, model performance was evaluated based on coefficient of determination (*R*^2^) and Spearman’s rank correlation coefficient (Spearman rho) between the predicted and measured values. In addition, the error between both values was estimated based on the root mean square error (RMSE) mean absolute error (MAE). A model that minimizes the error and maximizes the correlation coefficients was selected as best. For classification, model accuracy and confusion matrix, which gives an indication of sensitivity and specificity of the model, were evaluated. The classification model that maximizes test set prediction accuracy, sensitivity and specificity was selected as the best model.

Machine learning analysis was conducted in Python (ver. 3.6) using functions from the scikit-learn package (ver. 0.21.3). Analysis was performed using the Google’s Colaboratory platform, a freely available compute facility with in-built Python 3 kernel and machine/deep learning packages.

## Results

### Regression

**Figure 4:**
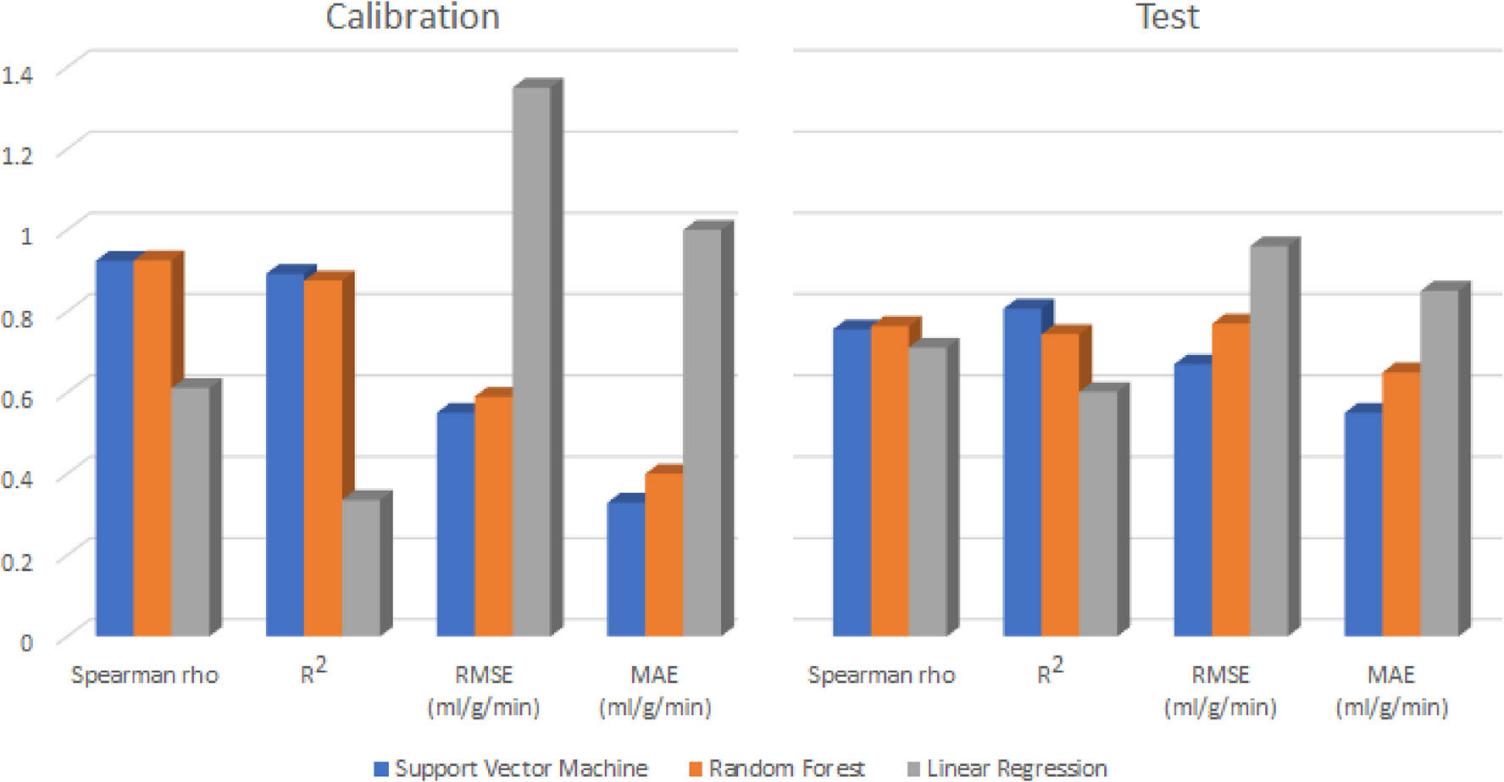
Comparison of the regression algorithms. The machine learning algorithms SVM and RF are superior compared with the linear regression method. Test set validation shows that SVM is the most accurate method to predict the value of MBF.

**Figure 5:**
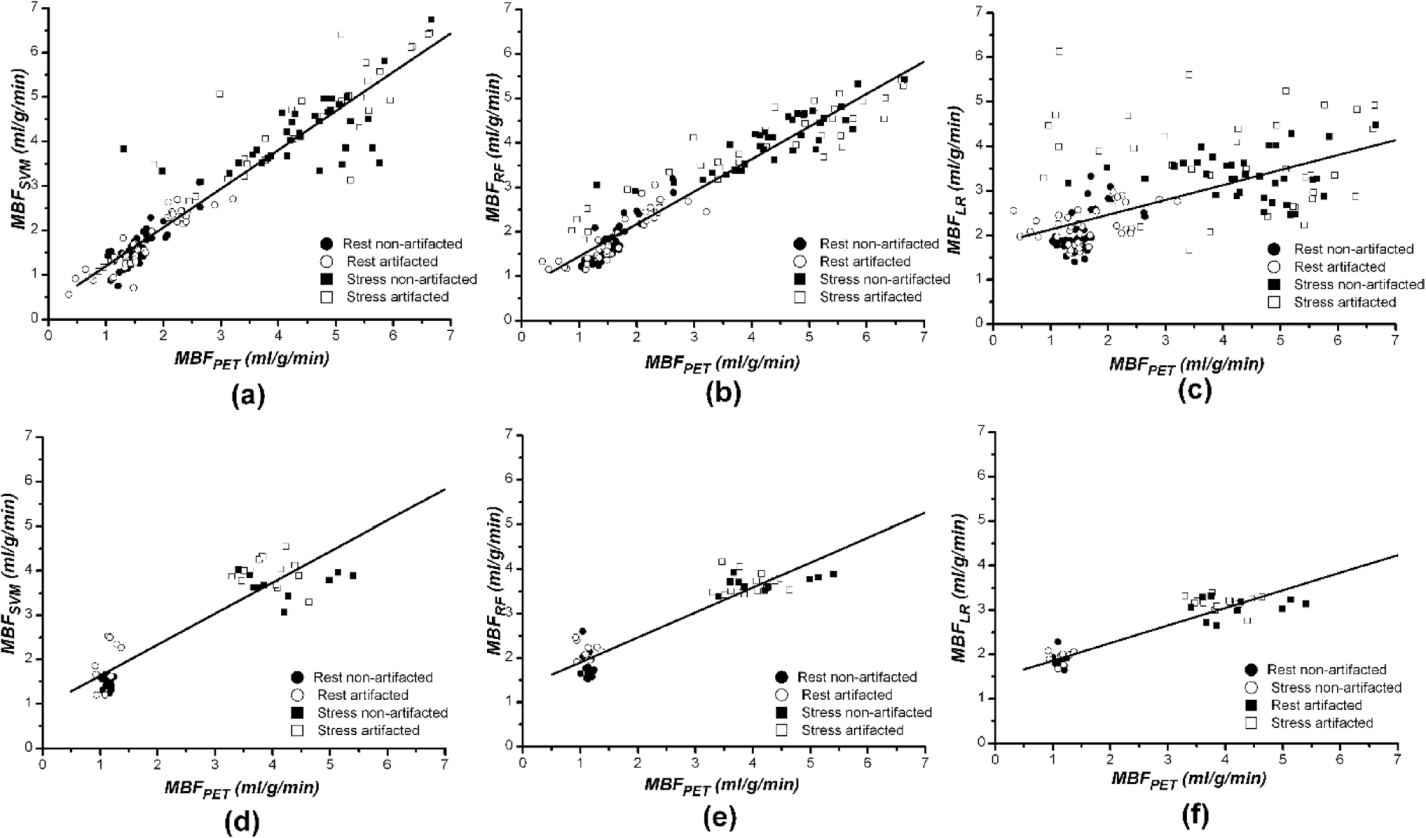
The calibration sets of (a) support vector machine, (b) random forest and (c) linear regression methods. The test sets of (d) support vector machine, (e) random forest and (f) linear regression methods. Large variation in test set of linear regression method can be seen. This is due to error, which is caused by dark rim artefact in numerous signals.

Regression model based on SVM (optimal parameters: *C *= 10, kernel = radial basis function, gamma = 100) was found to be optimal, with the lowest errors and highest correlations, for estimating MBF from tissue impulse response. While RF performed reasonably well in comparison with SVM, the model based on linear regression produced the weakest estimates of MBF. The performances metrics of the different regression algorithms for estimating MBF from the tissue impulse response signal are presented in Table 1 and visualized in Fig. 4. Remarkably high residual of the linear regression model can be observed in the calibration scatterplot of measured versus predicted MBF (Fig. 5c), particularly for samples with dark rim artefact-induced noise. However, the effect of artefact was minimal on the models based on SVM and RF, as they yielded reasonably accurate estimates of MBF also for samples with dark rim artefact (Figs. 5a and 5b).

**Table 1:** Performance metrics of the different regression algorithms evaluated for estimating myocardial blood flow (MBF) from tissue impulse response.

	Support vector machine	Random forest	Linear regression
Calibration			
Spearman rho	0.924	0.925	0.611
*R*^2^	0.892	0.875	0.336
RMSE (mL/g/min)	0.55	0.59	1.35
MAE (mL/g/min)	0.33	0.40	1.00
Test			
Spearman rho	0.756	0.764	0.711
*R*^2^	0.807	0.744	0.602
RMSE (mL/g/min)	0.67	0.77	0.96
MAE (mL/g/min)	0.55	0.65	0.85

*n*_calibration_ = 168 (*n*_non-artefact_ = 89 (53%), *n*_artefact_ = 79); *n*_test_ = 48 (*n*_non-artefact_ = 25 (52%), *n*_artefact_ = 23)

*Spearman rho* Spearman’s rank correlation coefficient, *R*^2^ coefficient of determination, *RMSE* root mean square error, *MAE* mean absolute error

### Classification

**Figure 6:**
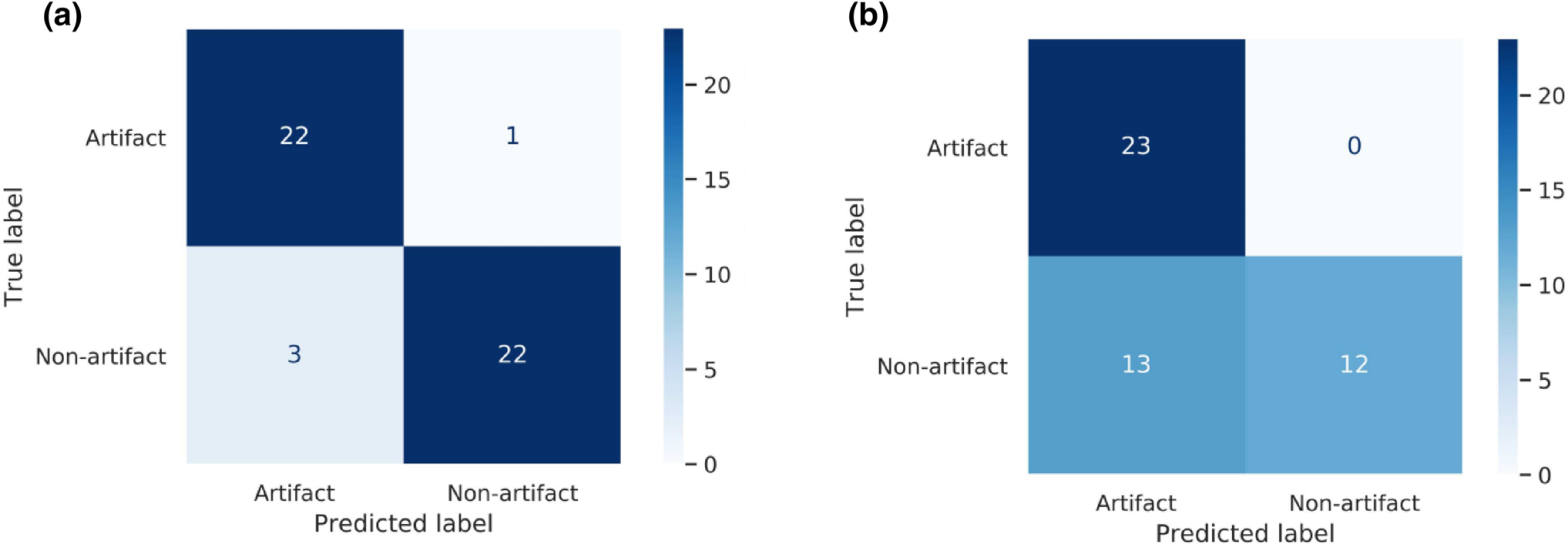
Confusion matrices showing the classification performance of (a) SVM and (b) RF for detecting if the tissue impulse responses contains artefact-induced noise.

In addition to prediction of MBF, classifiers were developed to evaluate the capacity of the SVM and RF to recognize samples with dark rim artefact-induced noise from their tissue impulse response. As in the regression analysis, SVM was optimal (optimal parameters: *C *= 1, kernel = radial basis function, gamma = 100) for detecting samples with dark rim artefacts based on their tissue impulse response signals (Fig. 6). While RF was sensitive in detecting artefacted signals, it misclassified 13 non-artefacted signals. Conversely, SVM classified only 4 out of 48 signals incorrectly as shown in the confusion matrices (Fig. 6).

## Discussion

In this study, we investigated the potential of machine learning methods for predicting myocardial blood flow (MBF) from the time-dependent tissue impulse response in a porcine model. The tissue impulse responses were obtained by modified dual bolus contrast agent-enhanced MRI examination.

In general, the machine learning methods (SVM and RF) were found to give more reliable estimates of MBF outperforming traditional linear regression (Figs. 5c and 5f), with SVM presenting the optimal model (Figs. 5a and 5d) for estimating the MBF from the impulse response input signal. It is worth noting that there was only slight difference in performance between SVM and RF in estimating the MBF from the impulse response input signal (Table 1 and Fig. 4). The dark rim artefact is very common with high field strengths and high contrast agent concentrations. This was the case also in this work. Dark rim artefact appeared as decreased SI in myocardium at the same time with the arrival of the contrast agent in the left ventricle. Distortion of the tissue enhancement curve causes incorrect results when determining the values of MBF in model independent deconvolution method, which produced the estimates of MBF for linear regression method. The model independent deconvolution method gets its estimate of MBF from tissue impulse response *h*(*t* = 0)^8^; however, the machine learning methods use the whole *h*(*t*), which includes multiple features, including noise introduced from dark rim artefact.

There are also other factors inducing noise to the tissue enhancement curve in clinical setting. For example, arrhythmias are very common in several myocardial diseases. Arrhythmias can disrupt the ECG-gating, which is necessary in dynamic heart imaging. Furthermore, dynamic imaging studies are often performed during free breath. As a result, it is possible that the heart moves during the dynamic imaging series. However, modern algorithms are able to correct the movements effectively. The uptake of contrast agent declines in ischemic myocardium, giving rise to reduced signal intensity in the affected areas. This leads to diminished signal-to-noise ratio of the tissue enhancement curve. In addition, all implants (coils, stents, *etc*.) in the heart may cause image artefacts, and thus noise to the tissue enhancement curve.

The relatively strong performance of SVM and RF in the classification and regression tasks defined in the present study, relative to linear regression, was expected, since these methods have previously demonstrated superior capacity in other areas of biomedicine.^18^ In addition, SVM and RF are capable of determining the relative importance of features within the input data, making them more robust and less sensitive to artefact-induced noise in the tissue impulse response input data. Therefore, machine learning methods can enable creation of models that are tolerant to artefact-induced noise. Thus, their ability to predict MBF reliably, even for artefacted parts of myocardium presents great clinical advantage.

As mentioned earlier, the image artefacts may lead to incorrect estimation of MBF. Therefore, it could be useful if the analysis method can recognize these distortions and inform the user. Machine learning methods, specifically SVM, was found to be sensitive (96%) and specific (88%) in distinguishing between artefacted and non-artefacted tissue impulse response input, with an accuracy 92% (Fig. 6a), outperforming RF which achieved a classification accuracy of 73% (Fig. 6b). The ability to tune the hyperparameters of the machine learning algorithms by automatically searching the hyperparameter space for the optimal hyperparameter combination allows tailoring of the algorithm to a specific dataset, thus improving the models’ performance. For example, while the linear kernel SVM is the most common, we determined via grid search of the hyperparameter search space that radial basis function, a nonlinear kernel, was optimal for defining the relationship between the input and output in both the classification and regression tasks.

In this study, MR and PET data collected from healthy and ischemic myocardium was used to create three different regression models. All the models developed in this study were based on data collected from pigs. Therefore, the method would need to be further validated and optimized prior to use with humans. The animal model of this study consisted only of five pigs, and therefore do not represent the full spectrum of myocardial conditions. Thus, we believe that the reliability and accuracy of support vector machine and random forest could be further improved with a larger study population.

Although PET was used as a reference for the contrast agent-enhanced MRI-based method, it should be noted that PET and contrast agent-enhanced MRI measure different things because, unlike radioactive water, the extraction of gadoteric acid is not 100%. However, the machine learning methods minimizes the discrepancy between these methods, because the developed machine learning models allows the prediction of PET perfusion from the tissue impulse response which is measured using gadoteric acid enhanced MRI.

In the future, PET will likely remain the gold standard. However, the availability of MRI is remarkably better than that of PET. More so, when compared with PET, MRI provides more information during the same imaging session. For example anatomy, pump function and tissue characteristics can be investigated during same MR imaging session. Furthermore, AI is a powerful and fast method for analysis of MR images. Thus, due to ease of accessibility, the technique presented in the present study could provide a potential alternative when PET is not available for all patients.

To conclude, machine learning methods enabled reliable identification of noisy signals and accurate estimation of MBF at rest and stress, even from noisy signals. Clinically, this could provide a tool for quality control in diagnostics and a protocol for easy estimation of MBF. SVM was found to give the most accurate estimates of MBF at both artefacted and non-artefacted areas of the myocardium. SVM was also optimal for detecting the artefacted data. The outcome of this study could constitute a significant improvement in the diagnosis of myocardial diseases. However, although pig is considered as excellent animal model for cardiology research, there are some anatomical differences between pig and human heart. Therefore, further research is needed to test the applicability of this technique for clinical use with human patients.
